# Supplementary material for: Pectoral herding: an innovative tactic for humpback whale foraging
Source: R Soc Open Sci. 2019 Oct 16;6(10):191104. doi: 10.1098/rsos.191104 (PMC6837203; doi:10.1098/rsos.191104)
Supplement: Supplementary file 1 [file rsos191104supp1.docx]

Supplemental Table 1. Observations associated with Whale A (#2360 in Southeast Alaska Humpback Whale Catalog) in Southeast Alaska (2016 to 2018). Type of behaviour, type of bubble-net, number of feeding sessions, presence (P) or absence (A) of pectoral herding and head tilt, number of feeding events with pectoral herding (NV means we observed pectoral herding but there were no photographs or videos from that day), and prey type(s) are shown for each date and location.

| Date | Location | Behaviour | Bubble-net Type | Number Feeding Sessions | Pectoral Herding | Number Events with Pectoral Herding | Number of Head Tilts  (P/A/UNK) | Prey Type |
| --- | --- | --- | --- | --- | --- | --- | --- | --- |
| 5/13/16 | Takatz Bay | Feeding | Solo | 1 | P | 4 | 1/2/1 | juvenile salmon |
| 5/14/16 | Takatz Bay | Feeding | Group | 1 | A |  |  | juvenile salmon |
| 5/20/16 | Warm Springs Bay | Feeding | Solo | 1 | P | 35 | 26/6/3 | juvenile salmon |
| 5/21/16 | Warm Springs Bay | Milling | N/A | 0 | N/A |  |  | N/A |
| 5/22/16 | Warm Springs Bay | Feeding | Solo | 1 | P | NV |  | juvenile salmon |
| 5/23/16 | Kelp Bay | Traveling | N/A | 0 | N/A |  |  | N/A |
| 5/24/16 | Warm Springs Bay | Feeding | Solo | 1 | P | 4 | 1/2/1 | juvenile salmon |
| 5/25/16 | Kelp Bay | Traveling | N/A | 0 | N/A |  |  | N/A |
| 5/28/16 | Kelp Bay | Traveling | N/A | 0 | N/A |  |  | N/A |
| 5/30/16 | Warm Springs Bay | Feeding | Solo | 1 | P | 3 | 0/3/0 | juvenile salmon |
| 6/8/16 | Kelp Bay | Feeding | Solo | 1 | P | 5 | 1/0/4 | juvenile salmon |
| 6/16/16 | Warm Springs Bay | Feeding | Solo | 1 | P | NV |  | juvenile salmon |
| 6/21/16 | Warm Springs Bay | Feeding | Solo | 1 | P | 2 | 0/0/2 | juvenile salmon |
| 5/17/17 | Kelp Bay | Feeding | Solo | 1 | P | 1 | 0/1/0 | juvenile salmon |
| 5/25/17 | Kelp Bay | Traveling | N/A | 0 | N/A |  |  | N/A |
| 5/28/17 | Kelp Bay | Traveling | N/A | 0 | N/A |  |  | N/A |
| 5/30/17 | Kasnyku Bay | Traveling | N/A | 0 | N/A |  |  | N/A |
| 5/31/17 | Kasnyku Bay | Feeding | Solo | 1 | P | 3 | 0/1/2 | juvenile salmon |
| 6/1/17 | Kasnyku Bay | Feeding | Solo | 1 | P | 45 | 26/17/2 | juvenile salmon |
| 6/6/17 | Kasnyku Bay | Feeding | Solo | 1 | P | 5 | 1/3/1 | juvenile salmon |
| 6/9/17 | Kasnyku Bay | Feeding | Solo | 1 | P | 5 | 1/4/0 | juvenile salmon |

Supplemental Table 1 (cont’d). Type of behaviour, type of bubble-net, number of feeding sessions, presence (P) or absence (A) of pectoral herding and head tilt, number of feeding events with pectoral herding (NV means we observed pectoral herding but there were no photographs or videos from that day), and prey type(s) are shown for each date and location.

| Date | Location | Behaviour | Bubble-net Type | Number Feeding Sessions | Pectoral Herding | Number Events with Pectoral Herding | Number of Head Tilts  (P/A/UNK) | Prey Type |
| --- | --- | --- | --- | --- | --- | --- | --- | --- |
| 6/10/17 | Kelp Bay | Feeding | Group | 1 | A |  |  | Pacific herring |
| 6/12/17 | Kelp Bay | Feeding | Group | 1 | A |  |  | Pacific herring |
| 6/28/17 | Kelp Bay | Feeding | Solo | 1 | P | 9 | 5/2/2 | Pacific herring or juvenile salmon |
| 7/8/17 | Kelp Bay | Milling | N/A |  | N/A |  |  | N/A |
| 7/15/17 | Point Wilson | Feeding | Group | 1 | A |  |  | Pacific herring |
| 5/22/18 | Kasnyku Bay | Feeding | Solo | 1 | P | 14 | 8/6/0 | juvenile salmon |
