# Supplementary material for: Pectoral herding: an innovative tactic for humpback whale foraging
Source: R Soc Open Sci. 2019 Oct 16;6(10):191104. doi: 10.1098/rsos.191104 (PMC6837203; doi:10.1098/rsos.191104)
Supplement: Observations associated with Whale B [file rsos191104supp2.docx]

Supplemental Table 2. Observations associated with Whale B (#2227 in Southeast Alaska Humpback Whale Catalog) in Southeast Alaska (16 May 2017). Time, location, light condition, number of feeding event, feeding behaviour, prey response, and details surrounding surface break are shown for each video. All feeding events involved a solo bubble-net.

| Video Title | Time | Location | Light Condition | Number of Feeding Event | Feeding Behaviour | Prey Response | Surface Break |  |
| --- | --- | --- | --- | --- | --- | --- | --- | --- |
| 05162017_01_lateral | 16:22 | NE | Sun | 1 | Lateral Lunge | N/A | head first | |
| 05162017_02_herd | 16:35 | SE | Sun | 2 | Pectoral Herding | N/A | left pectoral and head in same second | |
| 05162017_03_lateral | 16:38 | S | Shade | 3 | Lateral Lunge | N/A | left pectoral first | |
| 05162017_04_lateral | 16:46 | W | Shade | 4 | Lateral Lunge | N/A | Left pectoral first | |
| 05162017_05_herd | 16:59 | NE | Sun | 5 | Pectoral Herding | P | unknown | |
| 05162017_06_herd | 17:02 | NE | Sun | 6 | Pectoral Herding | P | left pectoral and head in same second | |
| 05162017_07_lateral | 17:12 | SW | Shade | 7 | Lateral Lunge | A | left pectoral and head in same second | |
| 05162017_08_vertical | 17:14 | SE | Shade | 8 | Vertical Lunge | A | head first | |
| 05162017_09_vertical | 17:29 | SE | Shade | 9 | Vertical Lunge | P | head first | |
| 05162017_10_herd | 17:37 | E | Sun | 10 | Pectoral Herding | P | left pectoral and head in same second | |
| 05162017_11_lateral | 17:57 | SE | Shade | 11 | Lateral Lunge | N/A | head first | |
| 05162017_12_lateral | 18:19 | E | Shade | 12 | Lateral Lunge | N/A | head first | |
| 05162017_13_vertical | 18:33 | NE | Shade | 13 | Vertical Lunge | N/A | head first | |
